# Single-Nucleotide Polymorphism (SNP) c.73G>A in the UBC9 (E2) SUMO Gene and Breast Cancer Risk in Polish Women

**DOI:** 10.3390/cancers18101616

**Published:** 2026-05-16

**Authors:** Hanna Romanowicz, Grzegorz Sychowski, Szymon Kalinowski, Szymon Sypniewski, Oleksandr Zakharin, Marek Zadrożny, Honorata Łukasiewicz, Dariusz Samulak, Beata Smolarz

**Affiliations:** 1 Laboratory of Cancer Genetics, Department of Pathology, Polish Mother’s Memorial Hospital Research Institute, Rzgowska 281/289, 93-338 Lodz, Poland; hanna-romanowicz@wp.pl (H.R.); grzegorz.sychowski@iczmp.edu.pl (G.S.); 2 Surgical Department, Regional Hospital, 99-400 Łowicz, Poland; szymon.kali@gmail.com (S.K.); szymon.sypniewski@interia.pl (S.S.); oloc.zakharin@gmail.com (O.Z.); 3 Department of Surgical Oncology and Breast Diseases, Polish Mother’s Memorial Hospital-Research Institute, 93-338 Lodz, Poland; marek.zadrozny@iczmp.edu.pl; 4 Faculty of Medicine and Health Sciences, Department of Nursing, The President Stanisław Wojciechowski Calisia University, 62-800 Kalisz, Poland; honorata.lukasiewicz@wp.pl; 5 Department of Obstetrics and Gynecology and Gynecological Oncology, Regional Hospital in Kalisz, 62-800 Kalisz, Poland; samulakd@wp.pl; 6 Department of Obstetrics, The President Stanisław Wojciechowski Calisia University, 62-800 Kalisz, Poland

**Keywords:** breast cancer, UBC9, gene, polymorphism

## Abstract

**Introduction**: Breast cancer is one of the major killers among malignant conditions worldwide, affecting one out of 10 women in industrialized countries and being the leading cause of cancer-related morbidity and mortality in women. The relationships between risk factors and breast cancer development are not exactly known. The selection of the *UBC9* gene and its c.73G>A polymorphism (rs11553473) in breast cancer studies is justified by the gene’s critical role in sumoylation, its impact on DNA repair, and its association with aggressive tumor characteristics. UBC9 (SUMO-conjugating enzyme) is frequently overexpressed in breast cancer, often 5–8-fold higher than in normal tissues, where it promotes tumor proliferation, invasion, and metastasis, often in a sumoylation-independent manner. Aim: In the present work, the association of polymorphism in the *UBC9* genes c.73G>A with breast cancer risk was investigated. **Materials and Methods**: In the reported study, paraffin-embedded tumor tissue was collected from women with lymph node-negative (*n* = 59) and lymph node-positive (*n* = 41) ductal breast carcinoma. Samples from age-matched, cancer-free women (*n* = 100) served as controls. The genotypes of the *UBC9* c.73G>A polymorphism were determined by ASO-PCR methods. **Results**: In the present work, a significant positive association between the *UBC9* c.73G>A G/A genotype and breast cancer is demonstrated. The variant A allele of *UBC9* increased breast cancer risk. Some correlation was observed between the genotypes of *UBC9* polymorphism and breast cancer invasiveness. A statistically significant increase was observed regarding G/A heterozygote frequency in stage II patients, according to Bloom–Richardson classification. There were no significant differences in genotype distribution among subgroups defined by TNM stage, histological grade, hormone receptor status, or HER-2 expression. **Conclusions**: In conclusion, the reported study indicates that the polymorphisms of the *UBC9* gene may be positively associated with the incidence of breast cancer. However, further research is needed on larger study populations.

## Simple Summary

The *UBC9* gene encodes the only known E2-conjugating enzyme for sumoylation, a post-translational modification crucial for cell cycle regulation and DNA damage response. Overexpression of UBC9, combined with genetic variations, can lead to genomic instability and increased breast cancer susceptibility. Studies indicate that carrying the variant A allele, specifically the G/A or A/A genotypes of UBC9 c.73G>A polymorphism, significantly elevates the risk of developing ductal breast cancer.

## 1. Introduction

Breast cancer is the most common malignant cancer in women in the world [1,2,3]. In addition to the well-known markers in the diagnosis of breast cancer, attention is paid to SUMO (small ubiquitin-like modifier) proteins, which may be important for the process of cancer transformation [4,5,6]. Through their action, SUMO proteins have an impact on many important life processes of the cell, such as DNA repair, the stability of various proteins and the response to cellular stress [7,8,9].

UBC9 is a key protein in the SUMO cycle, as it is the only conjugating enzyme, and so sumoylation can be inhibited by its elimination [10,11]. UBC9 is a protein that directly interacts with important DNA repair proteins, including RAD51 [12]. Although it is not a transcription factor itself, the UBC9 protein regulates the expression of many genes, both dependent and independent of the SUMO cycle [13,14]. On the other hand, many oncogenes and tumor suppressors, including MDM2, c-Myb, pRb, KAI1 and p53, are modified by SUMO proteins [15,16]. Androgen and estrogen receptors, which are involved in the development of prostate and breast cancers, are also sumoylated [17,18]. The role of the sumoylation process in the development and progression of cancer is evidenced by numerous studies showing changes in the expression of the genes encoding enzymes of the SUMO cycle (SAE1, SAE2, UBC9, SENP) in human cancer cells [19]. Overexpression of UBC9 (SUMO-coupling enzyme, the only known E2 enzyme for the sumoylation process) is characteristic of many cancers, including ovarian cancer, prostate cancer and lung adenocarcinoma [20,21]. The UBC9 protein is encoded by the gene of the same name, which is highly polymorphic. An association has been shown between the polymorphisms rs7187167, rs11248866 and rs8052688 of the *UBC9* gene and the histological grade of breast cancer [22,23,24]. The c.73G>A (Val25Met, rs11553473) polymorphism within the *UBC9* gene may be associated with histological differentiation of breast tumors and may be a useful prognostic marker in breast cancer [23]. Carriers of the variant A allele of the *UBC9* gene c.73G>A (Val25Met) polymorphism exhibit decreased DNA double-strand break (DSB) repair efficacy in breast cancer patients. This decreased repair, identified via neutral comet assay in studies like this one on ScienceDirect, is associated with higher levels of both endogenous and γ-irradiation-induced DSBs, highlighting the role of this variant in compromised genomic stability and potential breast cancer development [25].

The rs11553473 polymorphism is located in the N-terminal segment of *UBC9*, which participates in a non-covalent interaction with the SUMO protein [26]. Non-covalent UBC9–SUMO interactions involve the N-terminal helix of UBC9, as well as a loop between this helix and the first beta strand, β, which is partially used in the interaction with E1.

These non-covalent interactions of UBC9 and SUMO proteins are important for the formation of the SUMO chain. Valine 25 is the first amino acid of the beta strand of the UBC9 protein [27]. The mutation status at position 25 is as follows [28]:

- Trp25 and Arg25: Do not disrupt covalent bonds with SUMO proteins;
- Met25: The effects of this change are currently unknown (no research data).

Recent research points to a key role for DNA repair-related proteins in the development of breast cancer, particularly identifying the Cockayne syndrome group A (CSA) protein as a key contributor to cancer cell survival and chemoresistance.

A study by Filippi et al. demonstrated that targeting this pathway offers a promising strategy to improve treatment efficacy, especially for challenging subtypes like Triple-Negative Breast Cancer (TNBC) [29].

That research suggests that the CSA protein represents a very attractive target for the development of new specific anticancer therapies. By combining CSA inhibitors with standard chemotherapeutic agents, it may be possible to create more effective treatments for breast cancer with fewer and reduced likelihood of resistance [29].

Given the significant role of SUMO proteins in cancer transformation, it seems important to understand the potential role of the c.73G>A (Val25Met) (rs11553473) polymorphism of *UBC9* in the development of breast cancer.

## 2. Materials and Methods

### 2.1. Patients

**Figure 1:**
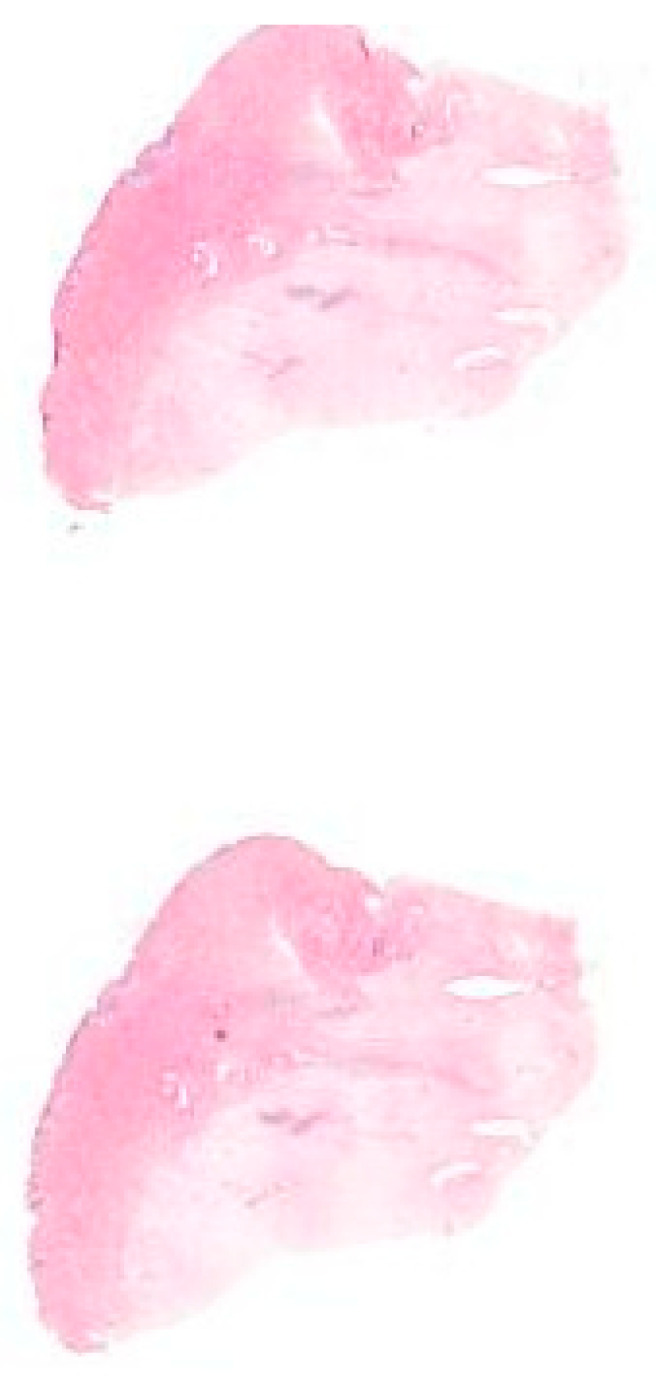
Carcinoma ductale invasivum (from Department of Pathology, Polish Mother’s Memorial Hospital Research Institute, Lodz, Poland). Image obtained from the scanner (Case Viewer 2.3, 3D Histech, Budapest, Hungary); magnification: 200×.

This study examined 100 patients with ductal breast cancer (carcinoma ductale) treated surgically at the Department of Surgical Oncology and Breast Diseases, Polish Mother’s Memorial Hospital Research Institute (PMMH-RI), in Lodz (Poland). The clinical diagnosis of cancer in each case was confirmed by an unambiguous cytological examination (fine-needle aspiration biopsy—BAC) or histopathological examination of the material from the coarse-needle biopsy. Some cases were identified intraoperatively by tumor freezing examination (“intra”). Accordingly, the primary surgical treatment, consisting of mastectomy or a sparing procedure, was undertaken. The material removed during the surgery was subjected to pathomorphological evaluation. Apart from classical morphological assessment, immunohistochemical tests were routinely performed to determine the expressions of progesterone estrogen receptors and HER-2. The stage of the disease was determined on the basis of the pTNM classification, and the histological type of the cancer was determined on the basis of the WHO classification (World Health Organization). The degree of histological malignancy was assessed according to the Bloom–Richardson classification. None of the patients had previously undergone chemotherapy or hormonal therapy. Histological examinations were carried out by experienced pathologists using a digital scanner of specimens and software for viewing them (Case Viewer 2.3, 3DHistech, Budapest, Hungary). Histological specimens were scanned using a Panoramic scanner (3DHistech, Budapest, Hungary) to obtain digital images (Figure 1).

In this study, the c.73G>A (rs11553473, Val25Met) polymorphism of the *UBC9* gene was analyzed in a group of women with breast cancer and in a control group without diagnosed breast and/or ovarian cancer, with no family history. Breast/ovarian cancer patients without family histories are less likely to carry germline (hereditary) mutations, making them an ideal reference point for identifying rarer genetic variants or variants with low penetrance. The control group consisted of 100 women. The material in this group was postoperative material embedded in paraffin from benign lesions. Patients were matched for age and gender (women).

The patients in the control group were aged 34–86 years (average age ± SD: 57 ± 10.54). The control group was consistent in age with the study group (age: 32–85 lat; average age ± SD: 59 ± 12.59).

This research used material collected in the archives of the Department of Clinical Pathomorphology of the PMMH-RI in the form of paraffin blocks containing sections from breast-cancerous tumors of patients operated on at the Department of Surgical Oncology and Breast Diseases, Polish Mother’s Memorial Hospital Research Institute. From each of the paraffin blocks, 2 pieces, 5 micrometers thick, and 2 mL of Eppendorf tubes were taken. Histopathological and genetic tests were carried out in the Department of Pathomorphology PMMH-RI. Patient characteristics are presented in Table 1. This study was approved by the Independent Bioethics Committee for Scientific Research at the Polish Mother’s Memorial Hospital Research Institute in Lodz (No. 51/2019).

### 2.2. DNA Isolation

DNA was isolated using the QIAmp DNA Mini Kit (Qiagen, Hilden, Germany) according to the manufacturer’s recommendations. The purity of the obtained DNA preparations was determined by the spectrophotometric method by measuring the absorbance of each sample twice at wavelengths of 260 nm and 280 nm. The accepted criterion of DNA purity was the A260/A280 value within the range of 1.8–2.0. DNA concentration was determined by the spectrophotometric method based on the absorbance value measured at a wavelength of 260 nm. This value corresponded to the following relationships:

1 OD = 50 μg DNA/mL

### 2.3. ASO-PCR Analysis

The ASO-PCR [allele-specific PCR] method was used to study the c.73G>A polymorphism of the *UBC9* gene. A 25 μL reaction mixture containing genomic DNA was used: 2.5 μL of PCR buffer; 0.5 μL dNTP (10 mM); 1 U of Taq polymerase (TaKaRa, Shiga, Japan); 1 μL each of suitable primers (Polgen, Łódź, Poland) and H_2_O (deionized). Amplification was carried out in the GeneAmp PCR system 9700 (Applied Biosystems, Waltham, MA, USA) thermocycler under appropriately selected conditions (Table 2).

The products of the ASO-PCR reaction were separated by electrophoresis in 2% agarose gel. The resulting image was interpreted according to Table 3.

### 2.4. Statistical Analysis

Statistical analysis of the distribution of genotypes and alleles in this study and the control group was carried out after prior confirmation that the obtained systems remained in a state of equilibrium according to the Hardy–Weinberg low. Genotype and allele distributions were analyzed, and their compliance with the Hardy–Weinberg distribution was assessed using the χ^2^ test. The differences between the distributions in the individual groups were also assessed using the χ^2^ test. The result was considered statistically significant, with a significance level, *p*, of less than 0.05.

Genotypes and alleles were assessed for their relationships to traits such as breast cancer risk using an odds ratio (OR) analysis and a 95% confidence interval (CI), which were calculated using a logistic regression model. We used Statistica v. 7.0 (StatSoft, Tulsa, OK, USA).

## 3. Results

The Hardy–Weinberg equilibrium was preserved in the *UBC9* rs11553473 polymorphism in all of the examined female patients (*p* > 0.050). Polymorphisms were observed in the linkage disequilibrium found between the analyzed groups of women (*p* ≤ 0.050).

Differences in the genotype distribution of the c.73G>A polymorphism of the *UBC9* gene were found between the breast cancer patient group and the control group (Table 4). In women with genotype G/A, the risk of developing breast cancer was 8.41 times higher compared to the control group (statistical result: significantly *p* < 0.0001). The A allele occurred with a statistically significantly higher frequency in the patients (42%) compared to the controls (24%). Its presence can more than double the risk of breast cancer.

Differences in the distribution of genotypes and alleles were found depending on the stage of cancer on the Bloom–Richardson grading system (I–III) (Table 5).

The frequency of the G and A alleles in the patients with stage I differs significantly from the frequency of alleles in the control group (*p* < 0.05). The presence of the A allele more than doubles the risk of stage I breast cancer.

The G/A genotype of the *UBC9* gene polymorphism occurred with a statistically significantly higher frequency (69%) in the stage I group compared to the controls. It can increase the risk of stage I breast cancer by more than six times.

**Figure 2:**
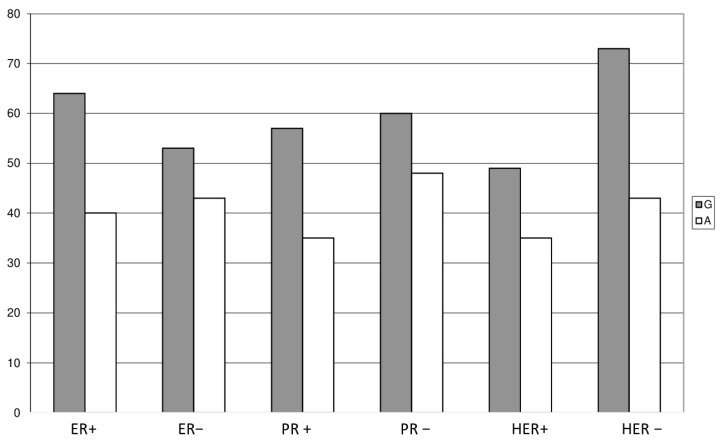
Distributions of genotypes and allele frequencies of the c.73G>A polymorphism in patients depending on the ER, PR and HER-2 status.

Differences in the distribution of genotypes and alleles have been shown depending on the stage of stage II breast cancer. The frequencies of the G and A alleles in patients with stage II differ significantly from the frequencies of the alleles in the control group (statistically significant differences, *p* < 0.05). The presence of the A allele more than doubles the risk of stage II breast cancer. The G/A genotype of the UBC9 gene polymorphism occurred with a statistically significantly higher frequency (69%) in the stage II group compared to the controls. It can increase the risk of stage II breast cancer by more than 15 times. Differences in the distribution of genotypes and alleles have been shown depending on the stage of stage III breast cancer. The presence of genotype G/A more than doubles the risk of stage III breast cancer. When comparing the distribution of genotypes and allele frequencies between the groups of three stages, statistically significant differences were noted only between the groups of stage II and III of the cancer. Heterozygote G/A may increase the risk of stage II cancer by more than 15 times. There was no association between the c.73G>A polymorphism and tumor size or metastases to surrounding lymph nodes, or progesterone and estrogen receptor and HER-2 status (Figure 2).

## 4. Discussion

The aim of this study was to determine the prevalence of polymorphic variants of the *UBC9* gene in breast cancer patients. An attempt was also made to assess the importance of the c.73G>A polymorphism of the *UBC9* gene as a prognostic and predictive factor for breast cancer in women.

Genetic variability, specifically single-nucleotide polymorphisms in the *UBC9* gene, has been associated with breast cancer risk, tumor grade, and prognosis [30,31]. Epidemiological studies and clinical analyses have confirmed a positive correlation between elevated levels of UBC9 and an increased risk of cancer, including the development and progression of breast cancer [10,32,33,34,35,36,37]. High expressions of UBC9 have been consistently associated with aggressive tumor phenotypes, including larger tumor size, higher tumor grade, and increased lymph node metastasis [38,39].

In studies of a population of Chinese women, it was shown that high expression of *UBC9* was associated with a poor response to chemotherapy and was a poor prognostic factor. In patients with low *UBC9* levels, the recurrence-free survival time was 6 years in 87% of the subjects; in women with high *UBC9* levels, the 6-year recurrence-free survival time was in only 68% of the patients [40]. The research of Moschos et al. showed that *UBC9* expression is elevated in colorectal and prostate cancer, while in metastatic breast cancer and lung cancer, it is reduced compared to normal tissues [40]. *UBC9* expression correlated with Dukes colorectal cancer staging. In the case of breast cancer, no relationship was found with the stage according to Bloom–Richardson. However, *UBC9* expression was higher in luminal-type breast cancers compared with other types, according to molecular classification [41]. Analyses by Wu et al. suggest that *UBC9* is overexpressed in breast, larynx, and lung cancers. The levels of UBC9 in these studies were more than 5 times greater in breast cancer cells than in normal cells. Researchers have suggested that UBC9 may undergo post-transcriptional regulation [36].The literature data indicate that UBC9, along with other SUMO proteins, such as PIAS1 and PIAS3, regulates the activity of hormone receptors such as the estrogen receptor (ERα) [42]. Genetic variability, specifically single-nucleotide polymorphisms in the *UBC9* gene (e.g., rs7187167), has been significantly associated with altered tumor characteristics, suggesting these variants contribute to differential UBC9 protein levels and, consequently, different clinical outcomes. This study involved a group of 100 women diagnosed with breast cancer and 100 without diagnosed breast pathological changes and with no family history of breast and/or ovarian cancer. The c.73G>A (Val25Met) polymorphism of the *UBC9* gene (rs11553473) was tested. This polymorphism was selected on the basis of the literature data cited earlier, which suggest a possible link between UBC9 polymorphisms and breast cancer in women [22,23,28]. Dünnebier et al. showed a relationship between the rs7187167 polymorphism of the *UBC9* gene and stage I of breast cancer [23]. This polymorphism was independent of tumor size and estrogen and progesterone receptor status.

The relationship between the c.73G>A (Val25Met) polymorphism of the *UBC9* gene (rs11553473) and the ability to repair DNA double-strand breaks in breast cancer patients was investigated [25]. The polymorphism under study may affect the effectiveness of DNA double-strand break repair in breast cancer patients.

Based on the study by Filippi et al., the Cockayne syndrome group A protein, a key component of Transcription-Coupled Nucleotide Excision Repair (TC-NER), is frequently overexpressed in breast cancer cells, including TNBC. This study supports the idea that cancer cells become “addicted” to high levels of CS proteins to withstand the stress of rapid division and DNA-damaging treatments. Furthermore, this work highlights that alterations in genome stability, potentially involving sumoylation pathways mediated by proteins like UBC9, are critical to this process of adaptation and progression [29].

The sumoylation process plays an important role in DNA crack repair by homologous recombination (HR). The key eukaryotic proteins of this repair pathway, RAD51 and RAD52, involved in the initial stage of this process directly interact with UBC9 and SUMO-1 [43]. The RAD51 protein is the most important protein in the HR process in mammals. In addition to it, the following proteins are involved in repair: RPA; RAD52; RAD51 paralogues RAD51B, RAD51C, XRCC2 and XRCC3;and proteins RAD54, RAD54B, BRCA1 and BRCA2 [44]. SUMO-1 can non-covalently bind to RAD51, thereby reducing cancer-cell resistance by inhibiting DNA double-strand break repair in the HR pathway [45]. Lowering the level of UBC9 interrupts the migration of RAD51 to the cell nucleus and thus inhibits its accumulation in repair clusters that are formed as a result of the action of the DNA-damaging factor [46]. Genotoxic stress induces the process of the sumoylation of another protein involved in DNA repair—BRCA1—at DNA damage sites [47]. BRCA1 activity as a result of sumoylation and its accumulation at DNA damage sites may be inhibited by certain *BRCA1* mutations predisposed to breast and ovarian cancer [48]. This study showed that cells with *BRCA1* mutations showed greater sensitivity to γ radiation than those without the mutations [49]. Research suggests that the *UBC9* c.73G>A polymorphism may have a similar effect [23]. It appears to be a potential marker for selecting the patients most sensitive to radiation therapy before qualifying them for this procedure. However, it is not an independent marker but is an additional factor among the group of previously detected markers. In studies, the presence of genotype G/A has reduced the ability to repair DNA [23]. No association has been found between the polymorphism and ER and PR receptor status. Genotype G/A was more common in HER-2 patients. As a result of detailed genetic analyses, the relationship between the c.73G>A polymorphism of the single-nucleotide proteins (SNPs) of the *UBC9* gene and the occurrence of breast cancer has been demonstrated in the current study.

In the presented studies, the cancer population and the control group were homogeneous in terms of gender (women) and matched in age. In the case of the c.73G>A polymorphism, the effect of genotype G/A on the risk of breast cancer was found. The studies were conducted in a group of breast cancer patients and in a control group. An increased incidence of genotype G/A was observed in the study group compared to the controls. Heterozygote G/A occurred with an eightfold higher frequency in the patients than in the controls. The obtained results indicate the possibility of a relationship between the c.73G>A polymorphism and the occurrence of this tumor.

Further study focused on the analysis of the c.73G>A polymorphism in women with different stages of breast cancer according to the Bloom–Richardson classification. In the group of women of stages I, II and III of cancer, a relationship was found between the c.73G>A polymorphism and this criterion. The frequency of genotype G/A in the study group differed significantly from the frequency of the alleles in the control group. It was higher compared to the controls. Also, the distribution of alleles was definitely different compared to the controls. In this study, there were no differences in the distribution of genotypes and c.73G>A alleles in the Polish population depending on tumor size; metastases to surrounding lymph nodes; and the statuses of estrogen, progesterone and HER-2 receptors.

The predictive value of the rs11553473 polymorphism for breast cancer depends largely on the patient’s ethnicity. The *UBC9* polymorphism as a risk marker is highest in European populations [22].

The ethnic distribution of the rs11553473 (UBC9 c.73G>A) polymorphism has revealed significant ethnic disparities in the minor allele A frequency, with key implications for breast cancer studies. The high prevalence in European populations, particularly within Eastern Europe (e.g., Polish populations), contrasted with its near-absence in African and East Asian populations, suggests that this marker, associated with a significantly elevated risk of ductal breast cancer (OR 6.86), is highly population-specific [22].

Key Ethnic Distribution Findings (rs11553473 A):

- Europeans: Highest frequency (5–10%), particularly in Eastern Europe;
- Admixed Americans (Latinos): Moderate frequency (2–4%);
- South Asians: Low frequency (1–3%);
- African/East Asian Populations: Very rare or absent (<1%) [50].

This sharp variation suggests the rs11553473 polymorphism likely emerged or was selected for post-exit from Africa. The high frequency in Europe, combined with the substantial association with breast cancer in this population, indicates a significant yet localized risk factor [22]. The suggestion to study haplotype diversity is valuable for determining if this variant is an ancient polymorphism or a relatively recent mutation that has risen in frequency due to genetic drift or adaptation in northern latitudes [51].

The sample size of 100 patients and 100 controls is very small for a genetic association study and represents a major limitation, as such studies often require hundreds or thousands of subjects to achieve sufficient statistical power (e.g., 80% power) to detect moderate effects. Small studies are often only capable of detecting very large genetic effects, missing more common, modest associations. With low power, a study may fail to detect a true genetic association, leading to the conclusion that no relationship exists when it actually does. Smaller samples are more sensitive to random variations and noise, which can produce spurious, statistically significant results (the “winner’s curse” or overestimation of effect sizes). Findings from a small sample are less likely to represent the broader population. The dataset is too small for multivariate analysis, which is a significant limitation.

A limitation of this study is that the control group consisted of patients with mild pathologies and not healthy people. This may have limited the generalizability of the results to the general population and potentially provided a conservative effect estimate. A control group consisting of women undergoing surgery for benign lesions is not standard. A healthy control population is an important and significant point in the design of clinical trials. Women with benign lesions are not entirely “normal”. Benign lesions may share inflammatory or genetic pathways with malignant breast tumors. These underlying conditions may be linked to the same genetic markers being tested for cancer, leading to an underestimation of the true genetic difference between the study group and the general population. It is worth noting that collecting tissue from completely healthy women from the general population is ethically difficult and rarely seen in invasive studies, which often forces researchers to use “hospital control”. Future studies should include healthy, age-matched patients to confirm molecular findings. The results are preliminary and require replication on much larger cohorts (e.g., from genomic consortia).

To sum up, in the presented work, a genetic analysis of the SNP polymorphism of the UBC9 gene was carried out in a population of Polish women in order to determine its significance for the development of cancer and to determine whether it is a risk factor for its occurrence. The c.73G>A polymorphism of *UBC9* was considered because the literature data indicate its greatest association with breast cancer. Studies of this type are very few in the world, and therefore this was the reason for the interest in this topic.

## 5. Summary

The presented work indicates that the c.73G>A polymorphism of the *UBC9* gene may be a risk factor for the development of breast cancer. However, this requires further work carried out on much larger groups of respondents. Furthermore, it cannot be ruled out that the influence of this polymorphism on cancer development is possible as a result of interaction with other factors. The process of cancer development is a complicated phenomenon and requires the comprehensive participation of many factors.

## Figures and Tables

**Table 1:** Characteristics of patients.

Breast Cancer	Patients (*n* = 100)	
Ductal breast cancer (*carcinoma ductale*) Bloom–Richardson grading system		
I	13	13
II	64	64
III	23	23
Tumor size		
T1 do 2.0 cm	69	69
T2 > 2.0–5.0 cm	30	30
T3 > 5.0 cm	1	1
T4 infiltrating the chest and skin	0	0
Lymph nodes		
N−	59	59
N+	41	41
ER		
Positive	52	52
Negative	48	48
PR		
Positive	46	46
Negative	54	54
HER-2		
Positive	42	42
Negative	58	58

**Table 2:** Primer sequences and thermal conditions for PCR reactions for a fragment of the *UBC9* gene.

Gene	*UBC9*
Polymorphism c.73G>A	G/A
Forward (5′→3′)	Primer G 5′-TTTCTTTTCTCTCAGGGTTTCG-3′ Primer A 5′-TTTCTTTTCTCTCAGGGTTTCA-3′
Reverse (5′→3′)	Primer 5′-TCTCAACCAGAAATGCCCTC-3′
Thermal amplification conditions	1. 95 °C—5 min (pre-denaturation) 2. 95 °C—30 s 3. 52 °C—60 s 4. 72 °C—60 s 5. 2 → 3 → 4 × 35 cycles 6. 72 °C—10 min (synthesis completion)

**Table 3:** Fragment lengths for different polymorphic variants of the *UBC9* gene.

Gene	Polymorphism	Lengths of the Obtained Fragments (bp)
*UBC9*	c.73G>A	G: 340 A: 340

**Table 4:** Distribution of genotypes and allele frequency of the c.73G>A polymorphism of the *UBC9* gene in breast cancer patients and control.

	Patients (*n* = 100)		Control (*n* = 100)		OR ^a^ (95% CI) ^b^	*p* ^c^
	Number	Frequency	Number	Frequency		
G/G	20	0.20	62	0.62	1.00 Ref.	
G/A	76	0.76	28	0.28	8.41 (4.32–16.35)	<0.0001
A/A	4	0.04	10	0.10	1.24 (0.35–4.39)	0.483
G	116	0.58	152	0.76	1.00 Ref.	
A	84	0.42	48	0.24	2.29 (1.49–3.52)	0.0002

^a^ OR, odds ratio; ^b^ 95% CI, confidence interval; ^c^ logistic regression model; *p* ≤ 0.050 is considered significant.

**Table 5:** Distributions of genotypes and allele frequencies of the c.73G>A polymorphism in patient and control groups.

	I/Control		OR ^a^ (95% CI) ^b^	*p* ^c^
	Number	Frequency		
G/G	3	0.23	1.00 Ref.	
G/A	9	0.69	6.64 (1.66–26.42)	0.005
A/A	1	0.08	2.06 (0.19–21.88)	0.472
G	15	0.58	1.00 Ref.	
A	11	0.42	2.32 (0.99–5.39)	0.078
	II/Control			
	Number	Frequency		
G/G	8	0.13	1.00 Ref.	
G/A	55	0.86	15.22 (6.40–36.17)	<0.0001
A/A	1	0.01	0.77 (0.08–6.88)	0.647
G	71	0.55	1.00 Ref.	
A	57	0.45	2.54 (1.57–4.09)	0.0002
	III/Control			
	Number	Frequency		
G/G	9	0.39	1.00 Ref.	
G/A	12	0.52	2.54 (1.571–4.09)	0.0002
A/A	2	0.09	1.37 (0.25–7.32)	0.498
G	30	0.65	1.00 Ref.	
A	16	0.35	1.68 (0.84–3.36)	0.188
	II/III			
	Number (Frequency)			
G/G	8 (0.13)		1.00 Ref.	
G/A	55 (0.86)		5.15 (1.65–16.10)	0.005
A/A	1 (0.01)		0.56 (0.04–7.44)	0.578
G	71 (0.55)		1.00 Ref.	
A	57 (0.45)		1.50 (0.74–3.03)	0.329

^a^ OR, odds ratio; ^b^ 95% CI, confidence interval; ^c^ logistic regression model; *p* ≤ 0.050 is considered significant.

## Data Availability

All data and materials, as well as software application, support the published claims and comply with field standards.
